# Oxygen Binding by Co(II) Complexes with Oxime-Containing Schiff Bases in Solution

**DOI:** 10.3390/ijms23105492

**Published:** 2022-05-14

**Authors:** Marek Pająk, Magdalena Woźniczka, Marta E. Lichawska, Bartłomiej Czerwiński, Jakub Włodarczyk, Jakub Fichna

**Affiliations:** 1 Department of Physical and Biocoordination Chemistry, Medical University of Lodz, Muszyńskiego 1, 90-151 Lodz, Poland; magdalena.wozniczka@umed.lodz.pl (M.W.); marta.lichawska@umed.lodz.pl (M.E.L.); 2 Department of Biochemistry, Medical University of Lodz, Mazowiecka 6/8, 92-215 Lodz, Poland; bartlomiej.czerwinski1@stud.umed.lodz.pl (B.C.); jakub.wlodarczyk@stud.umed.lodz.pl (J.W.); jakub.fichna@umed.lodz.pl (J.F.)

**Keywords:** Co(II) complexes, oxime-containing Schiff base, oxygen binding

## Abstract

The present work describes the complexation properties of two oxime-containing Schiff bases (used as ligands), viz. 2-hydroxyimino-N′-[1-(2-pyridyl)ethylidene]propanohydrazone (Hpop) and 2-hydroxyimino-N′-[(pyridine-2-yl)methylidene]propanohydrazone (Hpoa), with Co(II) ions in DMSO/water solution. Volumetric (oxygenation) studies were carried out to determine the uptake of molecular oxygen O_2_ in the formation of the complexes Co(II)-Hpop and Co(II)-Hpoa. The acquired data can be useful in the development of oxygen bioinorganic complexes of metal ions with Schiff base ligands in solution. Their properties allow them to be used as synthetic oxygen transporters. Moreover, the binding of dioxygen could play an important role in the research of catalytic activity by such systems.

## 1. Introduction

Interest in coordination compounds containing Schiff bases continues to grow due to their various biological implications (including antibacterial, antifungal, anticancer, antioxidant, anti-inflammatory, antimalarial, antiviral activity), and potential applications in designing new therapeutic agents. Attempts are also being made to determine their metabolism in living systems and the metal ion binding sites in metalloproteins.

Numerous attempts have been made to use Schiff base ligands and their complexes in biotechnology. They offer promise as catalysts in several reactions, such as polymerization, reduction of thionyl chloride, oxidation of organic compounds, the reduction reaction of ketones, aldol reaction, Henry reaction, epoxidation of alkenes, hydrosilylation of ketones, synthesis of bis(indolyl) methanes and the Diels–Alder reaction.

Previous studies have examined the oxygen absorption–desorption processes for square planar Mn(II), Co(II), and Ni(II) complexes of tetradentate Schiff base ligands; these were derived from the condensation reaction of ethylenediamine with salicylaldehyde, o-hydroxyacetophenone or acetyl acetone. In these cases, DMF (dimethylformamide) and chloroform were used as solvents. Cobalt(II) complexes showed significant sorption processes compared to Mn(II) and Ni(II) complexes. The presence of a pyridine axial base increases oxygen affinity [1,2,3,4].

The introduction of fragments of Schiff bases into the structure of (thia)calixarenes makes it possible to increase both their efficiency and selectivity regarding metal cations, such as Cu^2+^, Ni^2+^, Co^2+^, and Zn^2+^. Previous studies have synthesized Schiff bases with catechol fragments on thiacalix [4] arenes substituted at the lower rim in three conformations (*cone*, *partial cone*, and *1*,*3-alternate*). The obtained organic–inorganic copper-based materials, based on thiacalixarene-copper (II) complexes, have various applications, such as antifungal and antibacterial coatings, and as catalysts in the assembly of chemical and electrochemical sensors [5].

The complexation of thiosemicarbazone derivatives with Cu(II) ions improves their antitumor activity against melanoma cells. This activity is associated with DNA damage and cell cycle arrest in the G2/M phase, as well as disorders in antioxidant enzyme expression [6].

However, the generation of well-defined potential metallotherapeutics for cancer treatment presents a challenge. As such, their unique properties, and multiple possible pathways of action in cells, constitute an active area of research. More specifically, Schiff base ligands have been recognized as promising building blocks for the construction of stable and active complexes of numerous geometries and topologies. An example would be bimetallic [Ag_2_L_2_]^2+^ complex of Schiff base ligand L, which offers promise as a drug that can gain entry to harmful cancerous and inflammatory cells by binding with serum albumins and then inducing apoptosis [7].

**Figure 1:**
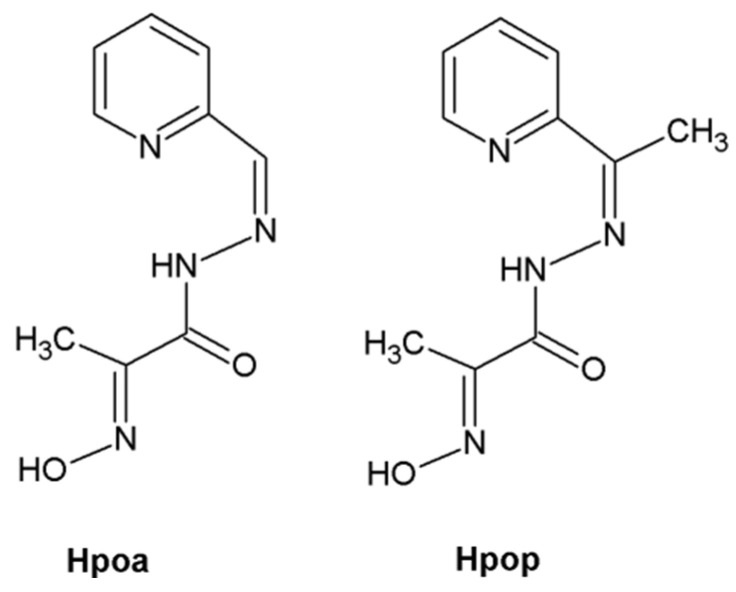
Structures of the ligands.

Our present study, based on previously validated potentiometric and spectrophotometric methods, indicates that the oxime ligands: 2-hydroxyimino-N′-[1-(2-pyridyl)ethylidene]propanohydrazone (Hpop) and 2-hydroxyimino-N′-[(pyridine-2-yl)methylidene]propanohydrazone (Hpoa) (Figure 1) exhibit a very high efficacy in the coordination of Co(II) ions. All measurements were conducted in 10/90 (*v/v*) DMSO/water solution [8,9,10].

## 2. Results and Discussion

Interestingly, at least three nitrogen donors were identified in the inner coordination sphere of the cobalt ion in Co(II)-Hpop and Co(II)-Hpoa [9] complexes; this suggests that these compounds follow Fallab’s 3N rule [11,12] and will be capable of dioxygen uptake.

**Figure 2:**
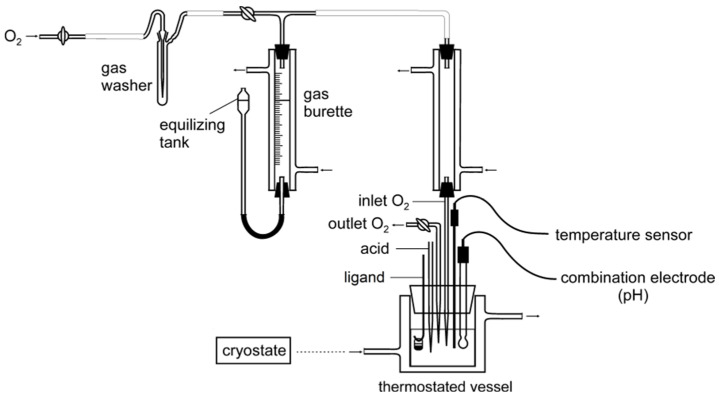
Laboratory set for volumetric–pehametric measurements.

**Figure 3:**
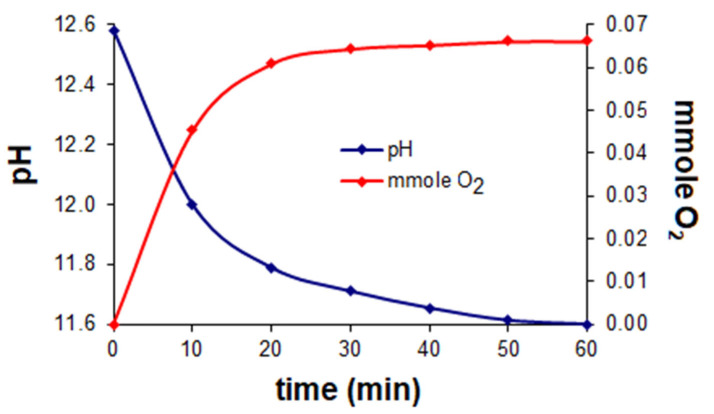
The Co(II)–Hpoa system at molar ratio 0.1:0.2 (mmol). Dependence of pH and numbers of mmole O_2_ bound during the oxygenation reaction at a temperature of ~0 °C.

**Figure 4:**
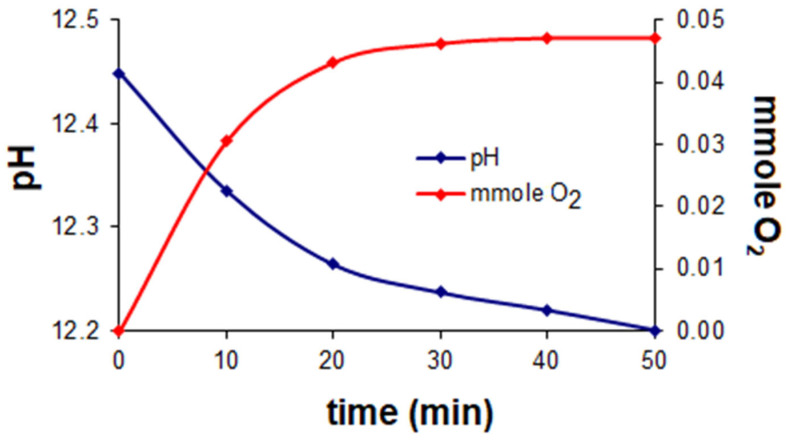
The Co(II)–Hpop system at molar ratio 0.1:0.2 (mmol). Dependence of pH and numbers of mmole O_2_ bound during the oxygenation reaction at a temperature of ~0 °C.

Simultaneous volumetric and pH-metric measurements in the presence of dioxygen were carried out by means of an isobaric laboratory set at ~0 °C (Figure 2). The pH was controlled by a precision PHM 85 pH-meter Radiometer (Copenhagen) with a GK 241C combination electrode. The sample volume was 30 cm^3^ in 3/27 (*v/v*) DMSO/water solution. The results indicated that the optimum ligand-to-metal concentration ratio was 2:1, cobalt concentration 3.3 × 10^−3^ M. Investigations of oxygenation of the Co(II)-Hpop and Co(II)-Hpoa systems indicated that dioxygen uptake is accompanied by a rise in pH to 11–12. The number of mmoles of bound dioxygen peaked at ~0.07 mmole per of 0.1 mmole cobalt (for Co(II)-Hpoa) (Figure 3) and ~0.05 mmole per of 0.1 mmole cobalt (for Co(II)-Hpop) (Figure 4).

Earlier potentiometric results for the Co(II)–Hpoa system suggest that only mononuclear bis species, viz. CoL_2_H_2_, CoL_2_H, CoL_2_, CoL_2_H_−1_ and CoL_2_H_−2_, are formed in DMSO/water solution across the full pH range [9]. The only two “active” complexes able to take up dioxygen are the forms CoL_2_H_−2_ (~90% of total cobalt) and CoL_2_H_−1_ (~10% of total cobalt), as they are the only ones present at pH ≥ 11, identified on the basis of complex species distribution as a function of pH.

For the Co(II)–Hpop system, the potentiometric results suggest the presence of mononuclear species, CoLH, CoL_2_H and CoL_2_H_−2_, and two dinuclear complexes, Co_2_L_2_H_−1_ and Co_2_L_2_H_−2_, in DMSO/water solution across the full pH range [9]. In the Co(II)–Hpop systems, the only “active” complexes able to take up dioxygen are CoL_2_H_−2_ (~60% of total cobalt) and Co_2_L_2_H_−2_ (~40% of total cobalt); these are the only ones present at pH ≥ 11, as indicated by complex species distribution as a function of pH.

Upon attachment of the oxygen moiety, the “active” species form an oxygen complex, with an O_2_^2−^OH (μ-peroxo-μ-hydroxo) (which exists in the pH range > 10) bridge between two cobalt ions formally oxidized to Co(III) [13]. The oxygen uptake reaction studied herein was practically irreversible, as demonstrated following acidification of the solution to pH~2.5 (the percentage of reversibility after acidification of the solution was in the range of 3–6%). Our dioxygen adducts may be regarded as an intermediate in the formation of a final, stable Co(III) complex. At low temperatures (−3 °C–0 °C), acidification of the solution results in protonation of the μ-peroxo-μ-hydroxo bridge to the μ-peroxo O_2_^2−^ (which exists within pH = 3–9), and eventually to the O_2_^2−^H^+^ (which exists in the pH range < 3). Moreover, at temperature ~0 °C and in the acidic medium, the O_2_^2−^ (μ-peroxo) bridge may be subsequently oxidized using strong oxidizers, e.g., Ce^4+^, MnO_4_^−^ or Cl_2_ ions. As a result, a stable paramagnetic complex is formed, with an irreversibly bound dioxygen moiety in the Co(III)-O_2_^−^-Co(III) (μ-superoxo) bridge [14].

Additionally, the oxygen-binding activity of other Schiff base complexes (bis-2-((1E)-((Z)-2-(1,2-diphenylethylideneamino) phenylimino)methyl)phenol, and bis-2-((1E)-((E)-2-(1-phenylethylideneamino)phenylimino)methyl)phenol with Fe(III), Co(II), and Ni(II) salts) was studied spectrophotometrically. Both were found to demonstrate significant oxygenation [15].

All O_2_ carriers (both natural and synthetic) fall into one of two categories: monomeric, with M:O_2_ = 1:1 stoichiometry, and dimeric, with M:O_2_ = 2:1 stoichiometry. It would be reasonable to assume that the dimeric structures would predominate, based on the theoretically estimated free standard Gibbs energy values of the O_2_ reactions between metal ions and their complexes. However, as a matter of fact, a much higher number of metals demonstrate a negative Δ*G*° value for dimer formation than for monomer formation [16]. This is a significant practical observation, because all the known dioxygen carriers form stable dimeric complexes in aqueous solution. Hence, it appears that the “active” complex is able to take up dioxygen: “active” complex + O_2_ = dimeric oxygen complex with an O_2_^2−^OH bridge, where: “active” complex = CoL_2_H_−2_, Co_2_L_2_H_−2_ (for the Co(II)–Hpop system), and CoL_2_H_−2_, CoL_2_H_−1_ (for the Co(II)–Hpoa system).

Our results are in line with recent studies conducted on similar ligands. The open-chain oxime ligands: N,N′-bis(2-hydroxyiminopropionyl)-1,2-aminoethane and N,N′-bis(2-hydroxyminopropionyl)-1,3-diaminopropane [17,18] were found to coordinate with Co(II) ions. Furthermore, in accordance with Fallab’s 3N rule [11,12] such complexes take up molecular oxygen in aqueous solution: a maximum of 0.45 mmoles of bound dioxygen was observed per 1 mmole of cobalt for H_2_pap, and 0.44 mmoles for H_2_pen [19]. The results are presented in Table 1.

Our data build on previous findings and can be useful in the development of new oxygen bioinorganic complexes of metal ions with Schiff base ligands in solution. Their properties allow them to be used as new synthetic oxygen transporters (e.g., in preservation fluids), just like the oxygen heteroligand systems of Co(II)–amino acid–imidazole [13]. Moreover, dioxygen binding will be valuable in further studies on catalytic activity by such systems. The next stage of research will be to apply the above-mentioned complexes as new heterogeneous cobalt nanocatalysts [20,21,22,23].

## 3. Materials and Methods

### 3.1. Reagents

2-hydroxyimino-N′-[1-(2-pyridyl)ethylidene]propanohydrazone and 2-hydroxyimino-N′-[(pyridine-2-yl)methylidene]propanohydrazone were synthesized by the Department of Chemistry, Kyiv National Taras Shevchenko University, Ukraine. Nitric (V) acid, p.a., P.O.Ch. Lublin; oxygen pure medical (99.7–99.8%); dimethyl sulfoxide ≥ 99.5%, Sigma-Aldrich. All solutions were prepared in double-distilled water.

### 3.2. Apparatus

The isobaric laboratory set for volumetric and pH-metric measurements (Figure 2) comprised a double-walled thermostated glass vessel of volume ca 80 mL, tightly closed with a silicon stopper. This vessel was equipped with a burette nozzle supplying the 4 M HNO_3_, GK 241C combination electrode, Radiometer Analytical 101 temperature sensor, gas inlet tube (dioxygen) connected with the gas burette, outlet tube, glass rod for hanging the glass vessel containing the small ligand, PHM 85 Precision pH-meter Radiometer (Copenhagen), Fisherbrand FBC 620 cryostat, Fisher Scientific, Electromagnetic Stirrer ES 21H (Piastów, Poland) and oxygen tank with reducing valve.

### 3.3. Oxygenation Reaction of the Co(II)–Hpoa and Co(II)–Hpop Systems

The thermostated vessel was filled with a solution containing an exactly weighed sample of the ligand in order to obtain a predicted Co(II)-ligand ratio. A small glass vessel with 0.1 mmoles of Co(NO_3_)_2_ was hung from a glass rod over the solution surface. The entire vessel was cooled to a temperature close to 0 °C to inhibit the irreversible oxidation of Co(II). After reading the initial pH and the initial volume level in the gas burette, the main experiment was started by inserting the Co(II) into the sample. The pH and dioxygen volume were noted in definite time intervals up to saturation. Over the course of the experiment, a rise in pH was observed, along with a change in color from entirely colorless to brown or even dark brown. At the end of oxygenation, which occurred when reaching pH ≈ 12, the solution was acidified to pH~2.5 with a small aliquot of 4 M nitric acid solution. The volume of dioxygen evolved concerning the total volume of dioxygen bound served as a measure of reversibility of oxygenation.

## Figures and Tables

**Table 1:** Uptake of O_2_ by the Co(II) systems (final value of pH, number of mmoles of O_2_ bound, percentage of reversibility). Ligand-to-metal molar ratio 2:1.

Co(II) Systems	pH	Mmoles O_2_	% Revers	Type of Bridge
Hpoa	11.600	0.0661	3.44	O_2_^2−^OH
Hpop	12.201	0.0472	6.30	
H_2_pap	~9.00	~0.45	<10.0	O_2_^2−^
H_2_pen	~9.00	~0.44	<10.0	

Hpoa—2-hydroxyimino-N′-[(pyridine-2-yl)methylidene]propanohydrazone. Hpop—2-hydroxyimino-N′-[1-(2-pyridyl)ethylidene]propanohydrazone. H_2_pap—N′-bis(2-hydroxyiminopropionyl)-1,3-diaminopropane. H_2_pen—N,N′-bis(2-hydroxyiminopropionyl)-1,2-aminoethane.

## Data Availability

Not applicable.
